# Resistance to Cotrimoxazole and Other Antimicrobials among Isolates from HIV/AIDS and Non-HIV/AIDS Patients at Bugando Medical Centre, Mwanza, Tanzania

**DOI:** 10.1155/2015/103874

**Published:** 2015-02-22

**Authors:** Karol J. Marwa, Martha F. Mushi, Eveline Konje, Paul E. Alele, Jeremiah Kidola, Mariam M. Mirambo

**Affiliations:** ^1^Department of Clinical Pharmacology, Weill Bugando School of Medicine, P.O. Box 1464, Mwanza, Tanzania; ^2^Microbiology and Immunology Department, Weill Bugando School of Medicine, P.O. Box 1464, Mwanza, Tanzania; ^3^Department of Community Medicine, School of Public Health, Catholic University of Health and Allied Sciences, P.O. Box 1464, Mwanza, Tanzania; ^4^Department of Pharmacology, Mbarara University of Science and Technology, P.O. Box 1410, Mbarara, Uganda; ^5^National Institute for Medical Research, P.O. Box 1462, Mwanza, Tanzania

## Abstract

Bacterial resistance has increased in the AIDS era and is attributed to the widespread use of cotrimoxazole prophylaxis against opportunistic infections in HIV/AIDS patients. In Tanzania, cotrimoxazole prophylaxis has been used for more than ten years. Little is known, however, about its impact on the spread of antibiotic resistance in HIV positive patients. This cross-sectional study was done to compare magnitude of bacterial resistance to cotrimoxazole and other antimicrobials among isolates from HIV infected patients on cotrimoxazole prophylaxis and those not on prophylaxis and non-HIV patients attending Bugando Medical Centre (BMC). Susceptibility testing on obtained urine and swab specimens followed Clinical Laboratory Standard Institute, 2010, Guidelines. Of 945 samples collected, 155 had positive bacterial growth after 24 hours of incubation. Of the positive samples (72), 46.4% were from HIV positive patients. The common isolates were *E. coli* 41.3% (64/155), *Klebsiella pneumoniae* 17.5% (27/155), and* Staphylococcus aureus* 16.1% (25/155). Overall, bacterial resistance to cotrimoxazole was 118 (76.1%); among isolates from HIV patients bacterial resistance was 54 (75%), and for isolates from HIV patients on prophylaxis bacterial resistance was 36 (81.3%). HIV seropositivity and cotrimoxazole prophylaxis are not associated with antibiotic resistance observed in bacteria infecting patients attending BMC, Mwanza, Tanzania.

## 1. Background

Multidrug resistance (MDR) bacteria like extended spectrum beta lactamase (ESBL) producers and methicillin-resistant* Staphylococcus aureus* (MRSA) are a major public health concern the world is facing [1, 2]. In developing countries where treatment options are limited MDR has led to increased morbidity and mortality [3]. Treatment of common bacterial infections like acute respiratory tract infections, urinary tract infections, wound infections, meningitis, and blood stream infections are very difficult when associated with MDR bacteria [4]. The problem is complex and is influenced by many interconnected factors such as poverty, self-medication, and misdiagnosis [3]. Chronic use of a specific antibiotic has been documented as the commonest factor that influences antibiotic resistance. This is due to constant exposure of bacteria to antibiotic pressure [2].

Cotrimoxazole has been used as a prophylactic agent against opportunistic infections in HIV/AIDS patients worldwide. In Tanzania, cotrimoxazole has been used as a prophylactic agent for over 10 years now and is used in all HIV and AIDS patients, starting with WHO stage 3, all adult persons with symptomatic HIV disease including HIV symptomatic pregnant women after the first trimester and before 37 weeks of pregnancy, and all children born of HIV positive women [5]. For non-HIV patients, in Tanzania, cotrimoxazole is used in diarrheal diseases [6]. Besides records of resistance reported before initiation of cotrimoxazole as a prophylactic agent, the chronic use of this drug among AIDS patients is expected to increase resistance due to selective pressure [7]. Previous studies have demonstrated a relationship between antibiotic consumption and the incidence of antimicrobial resistance in various bacterial infections [8–10].

A study undertaken in Soweto, South Africa, reported high resistance of* K. pneumoniae* and* S. aureus* to cotrimoxazole among isolates from HIV infected patients [11], with no data for HIV negative patients. The present study was carried out to determine the magnitude of bacterial resistance to cotrimoxazole and other antimicrobials among isolates from HIV infected patients on cotrimoxazole prophylaxis and those not on prophylaxes and non-HIV patients attending Bugando Medical Centre (BMC) in Mwanza, Tanzania.

## 2. Materials and Methods

This was a cross-sectional study conducted at microbiology laboratory of BMC from January to October 2012. This laboratory receives an average of 50 request forms for microbiological investigation of urine and pus samples per week. BMC is a consultant and teaching referral hospital for the Lake and Western zones of the United Republic of Tanzania. A systematic random sampling method was used whereby the laboratory investigation forms were assigned numbers daily; then all 1st, 5th, and 9th numbers were picked. Patients who were represented by these numbers were included in the study giving a total of 945 patients.

### 2.1. Laboratory Procedures

Urine and pus swab specimens were collected from enrolled participants. Specimens were processed as per standard operative procedures of the BMC laboratory adopted from the District Laboratory Practice manual in tropical countries [12]. Identification of bacteria was made by conventional physiological and biochemical methods [13, 14].

Antimicrobial susceptibility tests were done using the disk diffusion method (Kirby-Bauer's) according to Clinical and Laboratory Standards Institute (CLSI) [13] and WHO manual [14]. The antimicrobial discs tested include sulfamethoxazole/trimethoprim (1.25/23.75 *μ*g), ampicillin (10 *μ*g), erythromycin (15 mcg), ciprofloxacin (5 *μ*g), ceftriaxone (30 *μ*g), tetracycline (30 *μ*g), and amoxycillin/clavulanic acid (20 *μ*g). ESBL production was detected by using disc approximation method [15]. All these disks were obtained from Oxoid Hampshire (UK). Quality control was done using* Staphylococcus aureus* ATCC 25923 for gram positive bacteria and* Escherichia coli* ATCC 25922 for gram negative bacteria.

The results for the antimicrobial susceptibility test strain/bacteria (while checking that results of the quality control strain are within acceptable control range) were interpreted as susceptible (*S*) or resistant (*R*) by comparing the results to the CLSI 2010 standard zone diameter [13].

Patients whose HIV status was not known were counselled by a trained nurse from a care and treatment centre (CTC) and then each patient was requested to fill a consent form. HIV testing was done as per Tanzania National Algorithms [16].

*Ethical Issues.* The ethical clearance for conducting this study was obtained from Faculty of Medicine Research and Ethics Committee and the Institutional Review Board of Mbarara University of Science and Technology (MUST) and Joint CUHAS/BMC Research Ethical Committee (CREC). The patients were informed about study protocol and were requested to fill and sign an informed consent form.

## 3. Data Entry and Analysis

Data was entered on EXCEL (2007) data sheet and transferred to STATA 8 (Statistical Corporation, College Station, TX, US) for statistical analysis. Descriptive statistics for sociodemographic characteristics were obtained with respect to data type. Chi-square and Fisher's exact tests were performed for determining association between categorical variables where appropriate. Binary logistics regression was performed; odds ratios (OR) and 95% confidence intervals (95% CI) were reported as a measure of effect. *P* value of ≤0.005 was considered as statistically significant.

## 4. Results

### 4.1. Demographic Data of the Study Participants

**Figure 1:**
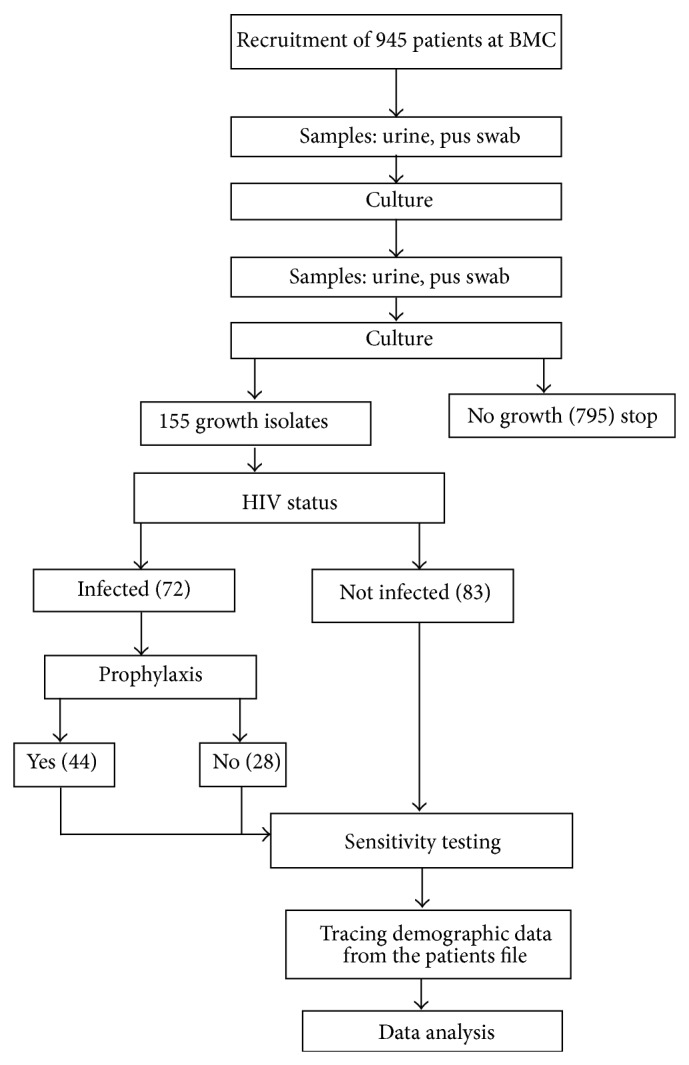
Study flow chart.

Of 945 patients' samples collected, 155 had significant bacteria growth and were analyzed. For analyzed samples (100) 64.5% were females. HIV positive patients were (72) 46.4% (Figure 1). Of HIV positive patients (44) 61% were on cotrimoxazole prophylaxis. Most of those who were not on cotrimoxazole prophylaxis were on WHO clinical stage 1, whereas most of those who were on cotrimoxazole prophylaxis were on stage 2, stage 3, or stage 4 (Table 1). Among HIV infected patients who were on cotrimoxazole prophylaxis, (31) 70.5% had CD4 count less than 350 whereas all of those not on prophylaxis had CD4 count greater than 350 (Table 1).

Majority of HIV patients on cotrimoxazole prophylaxis were on antiretroviral therapy (ART) (97.7%) while 67.9% of those not on cotrimoxazole prophylaxes were on ART (Table 1).

### 4.2. Bacteria Isolated

**Figure 2:**
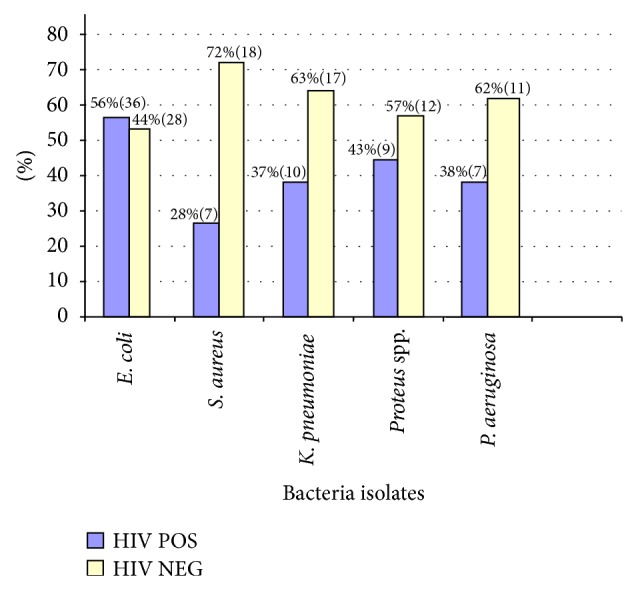
Distribution of bacteria isolates in relation to HIV status.

The study isolated both gram positive and negative bacteria dominated by* E. coli* 41.3% (64/155),* Klebsiella pneumoniae* 17.5% (27/155), and* Staphylococcus aureus *16.1% (25/155) (Figure 2).

In this study* E. coli *was mostly isolated from samples from HIV infected patients; samples from HIV negative patients showed a much higher percentage of other bacterial isolates like* S. aureus*. This study reveals higher resistance of* E. coli *to amoxicillin-clavulanic acid (43.6%), ampicillin (82.5%), tetracycline (78%), and cotrimoxazole (84.4%) (Table 2).

Of 155 samples with significant bacteria growth 72 (46.5%) were from HIV positive people. Bacteria resistance to cotrimoxazole on isolates from HIV infected patients was 54 (75%) and on those from HIV negative patients was 64 (77%) (*P* = 0.1). Similar results were observed with ampicillin, tetracycline, ceftriaxone, and ciprofloxacin (Table 3).

Of 72 isolates from HIV positive patients, 44 (61.1%) were from patients on cotrimoxazole prophylaxis. Data on susceptibility patterns of these bacteria are presented on Table 4.

### 4.3. Extended Spectrum Beta Lactamase Production

Of 155 bacteria isolates in this study 36 (23.2%) were ESBL producers. For isolates from HIV positive patients 23.6% (17/72) were ESBL producers. This study also found 44 isolates from HIV patients who were on cotrimoxazole prophylaxis, and, of them, 12 (27.3%) were ESBL producers (Table 5).

The study also noted that cotrimoxazole, amoxicillin-clavulanic acid, and ciprofloxacin resistance in bacteria can highly predict ESBL production. It was also found that being HIV positive and usage of cotrimoxazole prophylaxis do not predict ESBL production (Table 2). Resistance to cotrimoxazole and ciprofloxacin among ESBL producing bacterial isolates was significantly higher than that of non-ESBL producing bacterial isolates (*P* ≤ 0.001) (Table 5).

## 5. Discussion

The use of cotrimoxazole as an intervention to reduce the opportunistic infections in HIV positive patients is believed to highly reduce the morbidity and mortality in these patients [17]. UNAIDS recommends cotrimoxazole to be given to all HIV/AIDS patients in Africa [18], a practice which Tanzania has done for over 10 years now. However, selective pressure to bacteria due to constant exposure to antibiotic has been noted as the major contributor of antibacterial resistance developed by bacteria previously [2].

Despite the use of cotrimoxazole as prophylaxis in HIV positive patients, the current study detected that 46.4% of bacterial infection was from HIV positive patients. Of this 61% of bacteria were isolated from samples collected from HIV positive patients on prophylaxis. This might be either due to antibiotic resistance of these bacteria to cotrimoxazole or due to the WHO HIV stage, at which the use of prophylaxis agent is initiated in Tanzania.

Like it has been pointed out in other studies undertaken in Bugando Medical Centre [19–21] this study detected* E. coli* and* Klebsiella species* as the most common bacteria among gram negative bacteria and* S. aureus* as the commonest gram positive bacteria. In this study,* E. coli* resistance to cotrimoxazole among HIV positive patients suggests that HIV positive patients do not significantly harbor bacterial isolates with higher resistance to antimicrobials compared to HIV negative patients as seen from studies done elsewhere [22–24]. Similar findings were observed with* Klebsiella pneumoniae, *whereby there was no significant difference in bacterial resistance between HIV/AIDS patients and non-HIV patients to cotrimoxazole, amoxycillin-clavulanic acid, and ciprofloxacin, findings which are comparable with findings from other studies [23, 24]. Furthermore, the same results were observed with* Staphylococcus aureus *and* Proteus* species.

In our study we found a very high resistance to amoxicillin-clavulanic acid among ESBL producers (100%); this is comparable to a study done at the ICU Department of Muhimbili National Hospital of Tanzania [25] and is threatening, as it suggests that there might be an increase of ESBL producing bacteria in our community.

Hospitalization has been shown as a risk factor for acquisition of ESBL producing bacteria [26, 27]. In the current study, HIV negative patients are infected with more resistant* E. coli* isolates (67.9%) than HIV/AIDS patients (23.6%). This finding may be due to the fact that most HIV negative patients (75.5%) in our study were inpatients/hospitalized compared to HIV positive patients who were outpatients from Care and Treatment Center (CTC). Surprisingly, these results were not observed with* S. aureus, K. pneumoniae, *and* Proteus mirabilis.*

In this study, there was no significant difference in bacterial resistance to cotrimoxazole, ampicillin, tetracycline, and ciprofloxacin, between isolates from HIV/AIDS patients on cotrimoxazole prophylaxis, compared to those not on prophylaxis. This is similar to previous reports [28–30]. These findings can either suggest that majority of the bacterial pathogens have acquired resistance to cotrimoxazole due to selective pressure after more than ten years of this prophylactic practice, or cotrimoxazole prophylaxis is not a determining factor, since bacteria were resistant to cotrimoxazole long time ago [31].

Studies had established at the time of introducing cotrimoxazole prophylaxis more than ten years ago that cotrimoxazole prophylaxis reduces morbidity and mortality among HIV positive patients. The present situation may be different, as more than 80% of the bacteria causing opportunistic infections are resistant to cotrimoxazole.

*Pneumocystis jiroveci* which is the main target in cotrimoxazole prophylaxis among HIV/AIDS patients was not cultured in our laboratory. In this study, cross resistance between cotrimoxazole and other antimicrobials was not assessed. These may be limitations to the findings in our study.

*Recommendation.* Further studies are needed to assess the current morbidity and mortality in HIV positive patients taking prophylactic cotrimoxazole, in view of the findings that bacterial resistance to cotrimoxazole is high. Since the use of cotrimoxazole for prophylaxis in HIV positive patients is on-going as a policy of the government of Tanzania and many other countries, further studies are warranted with bigger participant populations to determine the prevalence of resistance to cotrimoxazole (and other antimicrobials) in these patients.

## Figures and Tables

**Table 1:** Background characteristics of 155 patients as categorized by HIV status.

Characteristics	Total (*n*)	HIV status	
		Negative	Positive
Sex	155		
Male		26 (47.3%)	29 (52.7%)
Female		57 (57.0%)	43 (43.0%)
Sample	155		
Urine		9 (15.2%)	50 (84.8%)
Pus swab		74 (77.1%)	22 (22.9%)
Gram stain	155		
Negative		66 (51.2%)	63 (48.8%)
Positive		17 (65.4%)	9 (34.6%)
Admission status	155		
Outpatients		6 (11.3%)	47 (88.7%)
In patients		77 (75.5%)	25 (24.5%)
WHO clinical stage	72		
1		NA	23 (32%)
2		NA	21 (29%)
3		NA	18 (25%)
4		NA	10 (14%)
CD4 count	72		
<350		NA	41 (57%)
>350		NA	31 (43%)
Cotrimoxazole prophylaxis	72		
Not on prophylaxis		NA	28 (39%)
On prophylaxis		NA	44 (61%)
Antiretroviral therapy			
Taking ART	62	NA	62 (83.3%)
Not taking	10	NA	10 (16.7%)

NA = not applied.

**Table 2:** Susceptibility data for common bacterial isolates to cotrimoxazole and other antimicrobials regardless of HIV or cotrimoxazole prophylaxis status.

	Resistant	Susceptible	*P*	Resistant	Susceptible	*P*
	*n*%	*n*%		*n*%	*n*%	
	Cotrimoxazole			Amoxycillin-clavulanic		
Bacteria isolates						
* E. coli *	54 (84.4)	10 (15.6)	0.1	27 (43.6)	35 (56.4)	<0.001
* S. aureus *	14 (56.0)	11 (44.0)		3 (13.0)	20 (87.0)	
* K. pneumoniae *	20 (74.1)	7 (25.9)		20 (83.3)	4 (16.7)	
* P. aeruginosa *	7 (87.5)	1 (12.5)		6 (75.0)	2 (25.0)	
* Proteus *	17 (80.9)	4 (19.1)		10 (47.6)	11 (52.4)	

	Erythromycin			Ceftriaxone		
Bacteria isolates						
* E. coli *	2 (50.0)	2 (50.0)		16 (48.5)	17 (51.5)	0.08
* S. aureus *	8 (57.1)	6 (42.9)		4 (30.8)	9 (69.2)	
* K. pneumoniae *	3 (60.0)	2 (40.0)		13 (72.2)	5 (27.9)	
* P. aeruginosa *	0 (0.0)	2 (100.0)		1 (25.0)	3 (75.0)	
* Proteus *	—	—		4 (33.3)	8 (66.7)	

	Ciprofloxacin			Ampicillin		
Bacteria isolates						
* E. coli *	20 (32.8)	41 (67.2)	0.49	47 (82.5)	10 (17.5)	0.001
* S. aureus *	3 (13.6)	19 (86.4)		3 (30.0)	7 (70.0)	
* K. pneumoniae *	6 (26.1)	17 (73.9)		21 (95.4)	1 (4.6)	
* P. aeruginosa *	1 (16.7)	5 (83.3)		6 (75.0)	2 (25.0)	
* Proteus *	5 (25.0)	15 (75.0)		16 (76.2)	5 (23.8)	

	Tetracycline			Cefuroxime		
Bacteria isolates						
* E. coli *	29 (78.4)	8 (21.6)	0.03	11 (64.7)	6 (35.3)	0.60
* S. aureus *	4 (44.4)	5 (55.6)		3 (37.5)	5 (62.5)	
* K. pneumoniae *	19 (95.0)	1 (5.0)		10 (71.4)	4 (28.6)	
* P. aeruginosa *	5 (100.0)	0 (0.0)		2 (66.7)	1 (33.3)	
* Proteus *	14 (82.4)	3 (17.6)		5 (45.5)	6 (54.5)	

**Table 3:** Antimicrobial resistance patterns based on HIV status.

Antimicrobial agent	HIV status		*P*	Crude
	Negative	Positive		
	*N* (%)	*N* (% )		OR (95% CI)
Cotrimoxazole				
Resistant	64 (77.1)	54 (75)	0.76	1
Susceptible	19 (22.9)	18 (25)		0.89 (0.42–1.87)
Amoxycillin-clavulanic acid				
Resistant	47 (60.0)	23 (34.0)	0.02	1
Susceptible	32 (40.0)	45 (66.0)		0.35 (0.17–0.70)
Ciprofloxacin				
Resistant	14 (19.0)	22 (33.3)	0.07	1
Susceptible	58 (81.0)	44 (66.7)		2.07 (0.94–4.56)
Ampicillin				
Resistant	49 (72.1)	51 (86.4)	0.06	1
Susceptible	19 (27.9)	8 (13.6)		2.47 (0.97–6.28)
Ceftriaxone				
Resistant	21 (42.9)	6 (66.7)	0.59	1
Susceptible	28 (57.1)	3 (33.3)		1.5 0 (0.33–6.81)
Tetracycline				
Resistant	51 (82.3)	26 (81.3)	0.91	1
Susceptible	11 (17.7)	6 (18.7)		0.94 (0.31–2.83)
Erythromycin				
Resistant	11 (64.7)	3 (27.3)	0.06	1
Susceptible	6 (35.3)	8 (72.7)		0.21 (0.03–1.26)

**Table 4:** Susceptibility data for isolates among HIV patients on prophylaxis and those not on prophylaxis.

	Cotrimoxazole prophylaxis		*P*	Crude
	Not on prophylaxis	On prophylaxis		
	*N* (%)	*N* (%)		OR (95% CI)
Cotrimoxazole				
Resistant	18 (64.3)	36 (81.8)	0.09	1
Susceptible	10 (35.7)	8 (18.2)		2.5 (0.82–7.65)
Amoxycillin-clavulanic acid				
Resistant	11 (40.7)	12 (29.3)	0.33	1
Susceptible	16 (59.3)	29 (70.7)		0.60 (0.21–1.69)
Ciprofloxacin				
Resistant	8 (33.3)	14 (33.3)	1.00	1
Susceptible	16 (66.7)	44 (66.7)		1 (0.34–2.92)
Ampicillin				
Resistant	19 (86.4)	32 (86.5)	0.99	1
Susceptible	3 (13.6)	5 (13.5)		2.47 (0.97–6.28)
Tetracycline				
Resistant	14 (82.4)	12 (80.0)	0.87	1
Susceptible	3 (17.6)	3 (20.0)		0.86 (0.14–5.21)

**Table 5:** Predictors of ESBL producing isolates among 155 clinical isolates.

	ESBL nonproducer	ESBL producer	*P*	Crude
	*n*%	*n*%		OR (95% CI)
Cotrimoxazole				
Resistant	83 (69.8)	35 (97.2)	0.001	1
Susceptible	36 (30.2)	1 (2.8)		15 (1.85–125 .5)
Amoxycillin-clavulanic				
Resistant	35 (31.3)	35 (100)	<0.001	NA
Susceptible	77 (68.7)	0. (0.0)		
Ciprofloxacin				
Resistant	14 (12.9)	22 (75.9)	<0.001	1
Susceptible	95 (87.1)	7 (24.1)		0.05 (0.01–0.13)
Ampicillin				
Resistant	71 (72.5)	29 (100)	0.001	NA
Susceptible	27 (27.5)	0 (0.0)		
Tetracycline				
Resistant	47 (73.4)	30 (100)	0.002	NA
Susceptible	17 (26.6)	0 (0.0)		
Ceftriaxone				
Resistant	19 (47.5)	15 (83.3)	0.01	1
Susceptible	21 (52.5)	3 (16.7)		5.52 (1.26–24.4)
HIV status				
Negative	64 (53.8)	19 (52.8)	0.92	1
Positive	55 (46.2)	17 (47.2)		1.04 (0.49–2.19)
Cotrimoxazole prophylaxis				
No	23 (41.9)	5 (29.4)	0.36	1
Yes	32 (58.1)	12 (70.6)		1.72 (0.53–5.57)
